# Supplementary material for: Self-interaction of NPM1 modulates multiple mechanisms of liquid–liquid phase separation
Source: Nat Commun. 2018 Feb 26;9:842. doi: 10.1038/s41467-018-03255-3 (PMC5827731; doi:10.1038/s41467-018-03255-3)
Supplement: Supplementary file 1 — Supplementary Information [file 41467_2018_3255_MOESM1_ESM.pdf]

SELF-INTERACTION OF NPM1 MODULATES MULTIPLE MECHANISMS OF LIQUID-LIQUID PHASE SEPARATION

Mitrea *et. al*.

Supplementary Figures

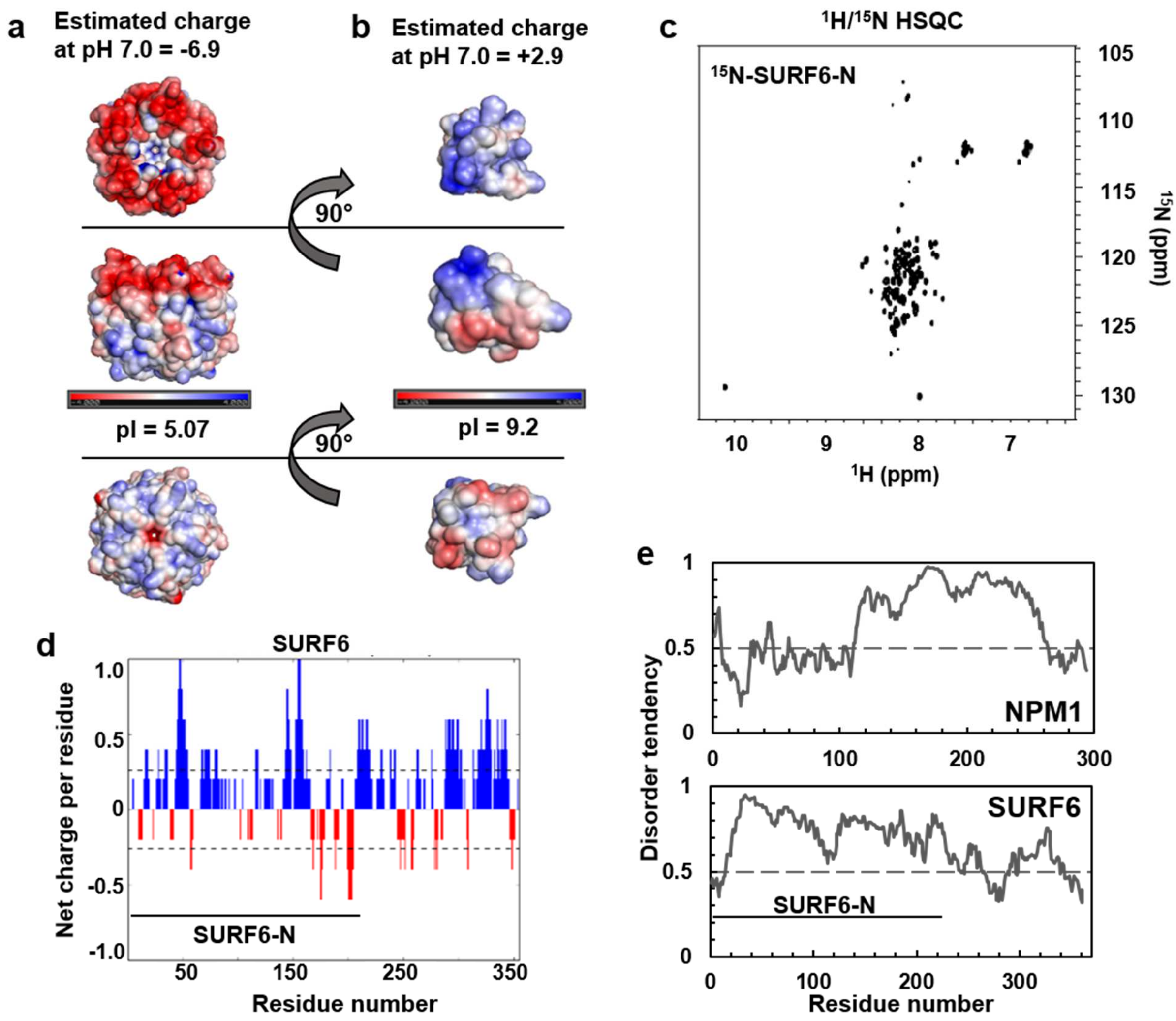

Supplementary Figure 1: **NPM1 and SURF6 sequence and structural features (a)** APBS surface electrostatic map of the N-terminal OD of NPM1 ($NPM1^{OD}$; PDB ID 4N8M) and **(b)** C-terminal domain of NPM1 ($NPM1^{C54}$; PDB ID 2VDX); **(c)** $^{1}H/^{15}N$-HSQC NMR spectrum of ^{15}N-SURF6-N; **(d)** Pappu-Das diagram of net charge per residue for full length SURF6 (http://pappulab.wustl.edu/CIDER/analysis); **(e)** IUPRED (http://iupred.enzim.hu) disorder prediction for NPM1 and SURF6.

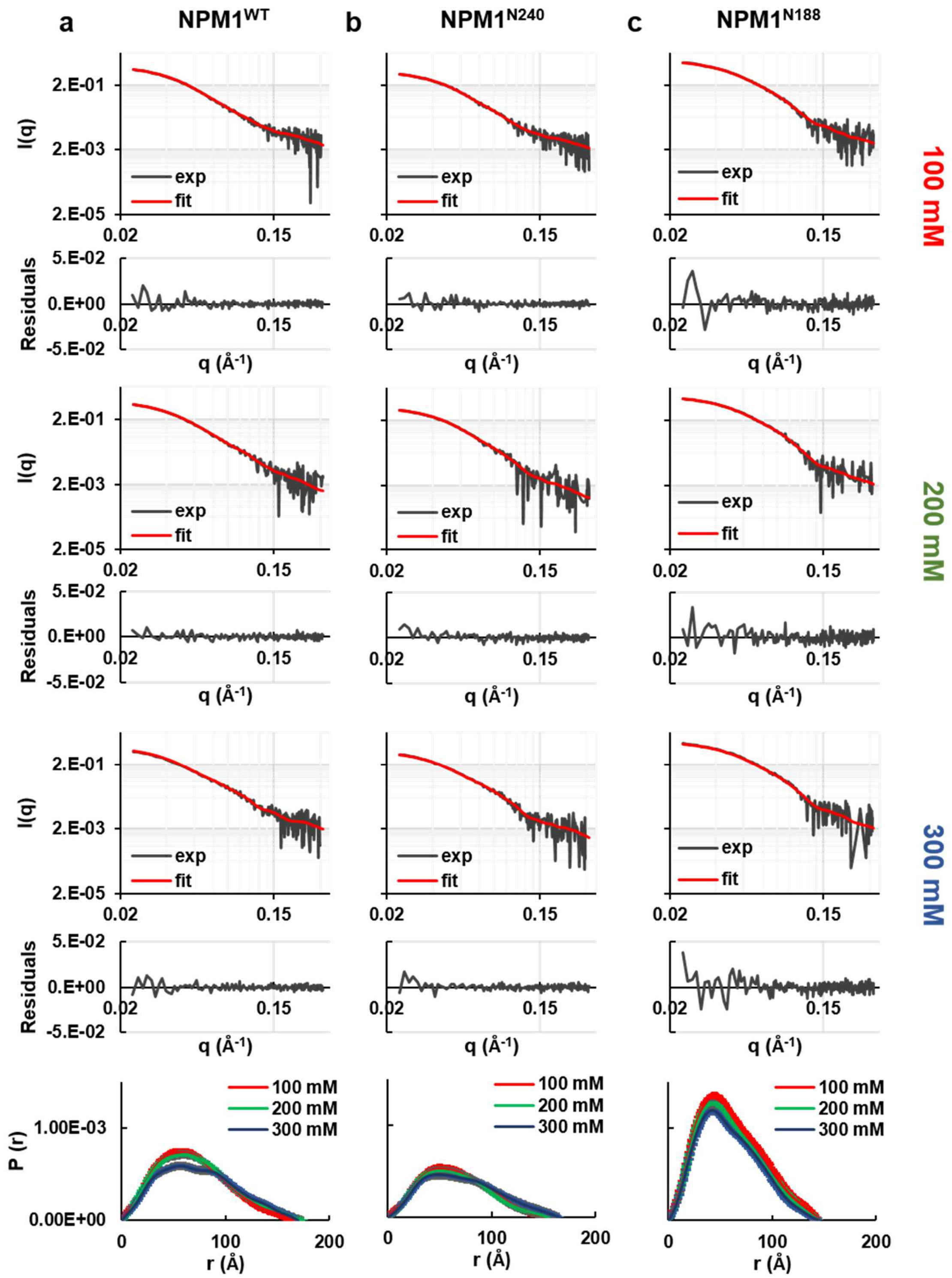

Supplementary Figure 2 Ionic strength modulates the conformational ladscape of NPM1 constructs with intact IDR Representative GNOM and pair-wise distance distribution, P (r), curve fits of the SAXS data used for R_g determination, as a function of ionic strength; **(a)** $NPM1^{WT}$, **(b)** $NPM1^{N240}$, **(c)** $NPM1^{N188}$.

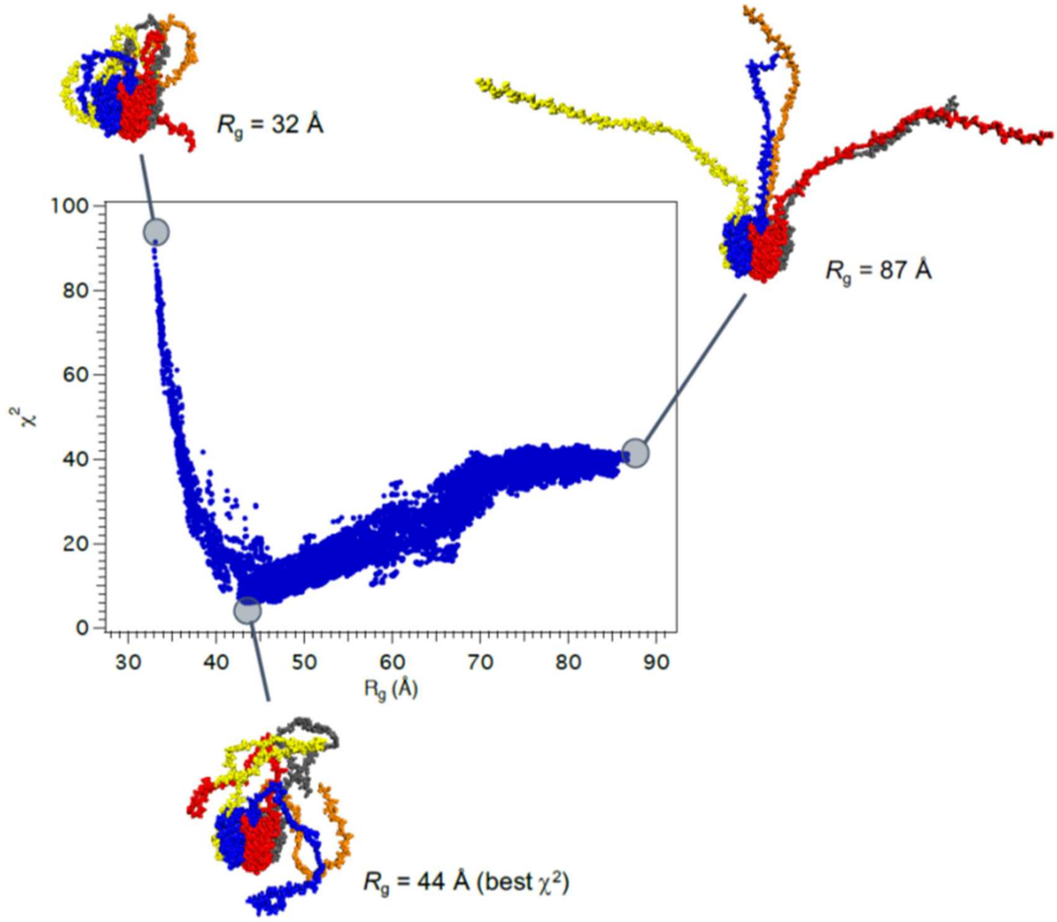

Supplementary Figure 3 Goodness of fit plot of individual NPM1^{N188} molecular models *vs.* experimental scattering curves SASSIE modeling of NPM1^{N188} supports the hypothesis that this construct adopts an ensemble of partially expanded conformations, due to the electrostatic repulsion within its truncated IDR.

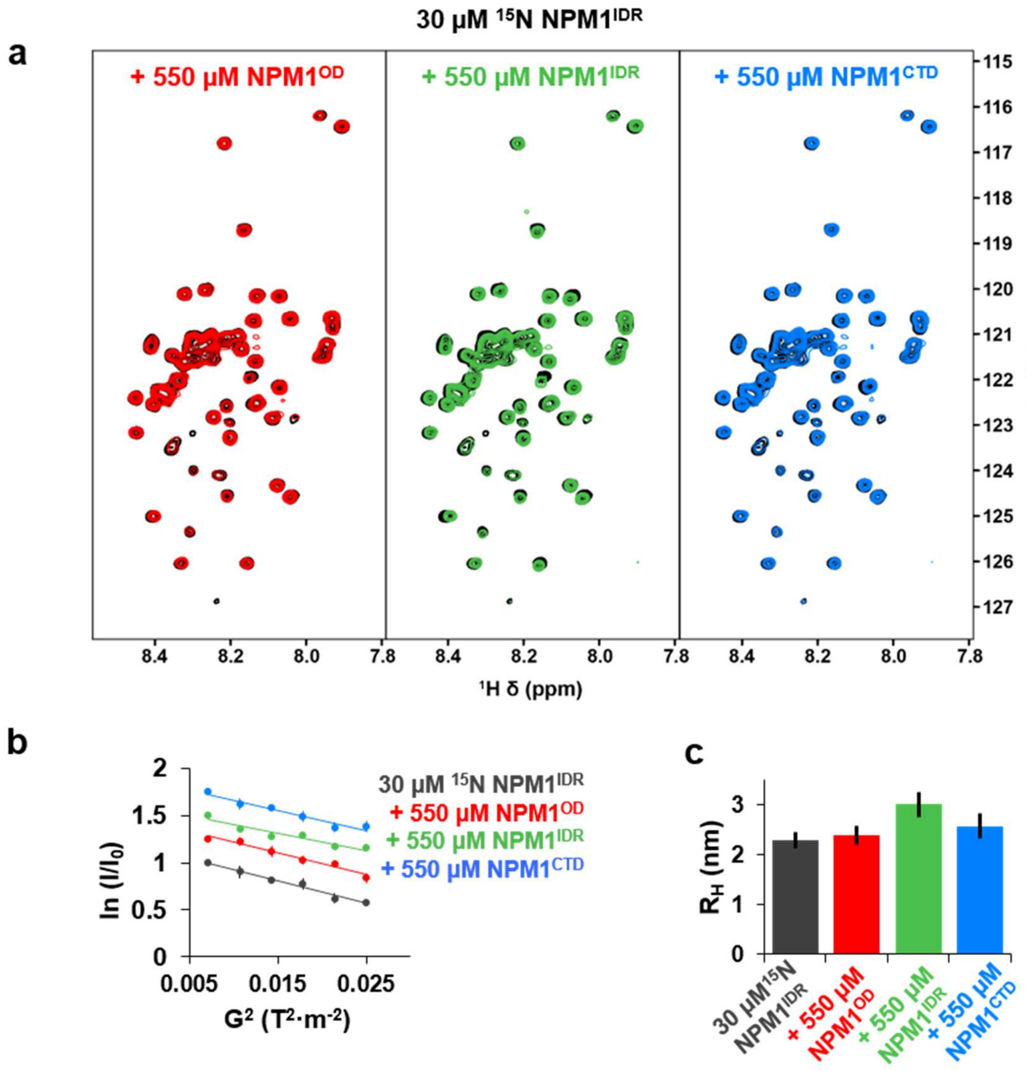

Supplementary Figure 4 IDR interacts with itself, but not with the folded domains in NPM1 NMR spectroscopy showed that the IDR of NPM1 weakly interacts with itself but does not interact with either folded domain. **(a)** $^{1}H/^{15}N$ HSQC spectra for 30 μM ^{15}N NPM1^{IDR} in the presence of excess non-isotope-labeled NPM1^{OD}, NPM1^{IDR} and NPM1^{CTD}; **(b)** ^{15}N-filtererd diffusion curves for 30 μM ^{15}N NPM1^{IDR} in the absence (grey) and presence of 550 μM oligomerization domain (NPM1^{OD}, red), IDR (NPM1^{IDR}, green), and CTD (NPM1^{CTD}, blue); values represent mean ± s.d.; n = 3. **(c)** Changes in R_H values of ^{15}N NPM1^{IDR} derived from the ^{15}N-filtered diffusion experiments (see also Supplementary Table 3); errors in the diffusion constant are the standard error to the fit from all 18 data points from (b).

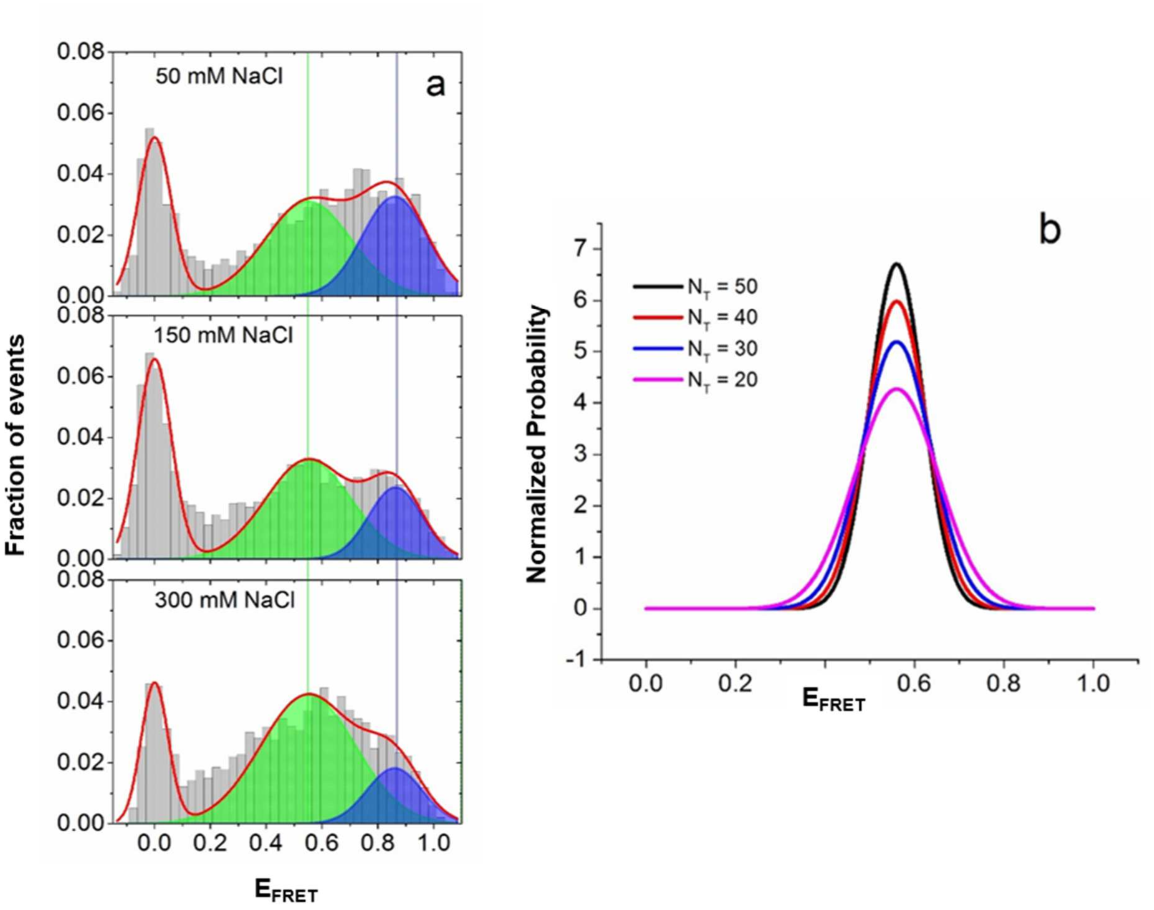

Supplementary Figure 5 smFRET analysis of NPM1 molecular expansion under high ionic strength conditions (a) Two-peak fitting (solid lines) of the NPM1 smFRET data (from Fig. 2e in the *main text*) at various [NaCl] using a Gaussian model. The peak at zero is due to molecules lacking an active acceptor dye. **(b)** Shot-noise simulations at different threshold conditions (at 300 mM NaCl) showing the variation of the peak width due to the Poissonian distribution of photons. Note that the experimental N_T is ≥ 40 in our experiments.

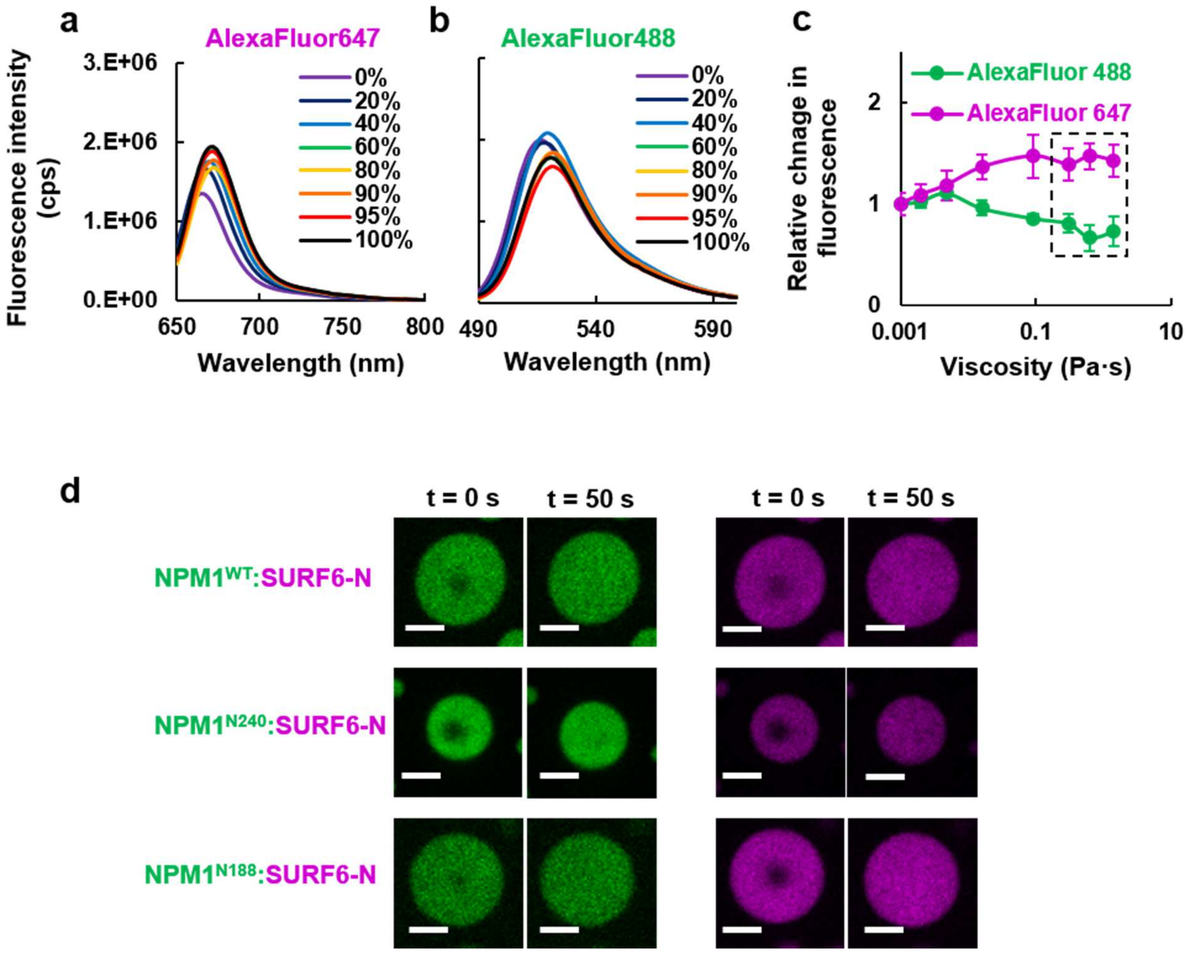

Supplementary Figure 6 Quantum yield changes of the fluorescent dyes used in the study, as a function of increased viscosity. Emission spectra of AlexaFluor 647 **(a)** and AlexaFluor 488 **(b)** in water, containing the specified percentage of Glycerol; **(c)** emission intensity integrated over the range of the microscope's bandpass filters, normalized to the integrated intensity of each AlexaFluor dye dissolved in water; values represent mean ± s.d.; n = 3; the dotted box highlights the points used to calculate the emission fluorescence correction factors. Corresponding viscosity values were determined using http://www.met.reading.ac.uk/~sws04cdw/viscosity_calc.html; **(d)** representative images of 20 μM NPM1-constructs : 20 μM SURF6-N droplets recovering from photobleaching; AlexaFluor 488-labeled NPM1 constructs are shown in green and AlexaFluor 647-SURF6-N is shown in magenta; scale bar = 5 μm.

Supplementary Tables

Supplementary Table 1 Isoelectric points and estimated charges of full length and segments of NPM1. Estimates were obtained using Protein Calculator v3.4 (http://protcalc.sourceforge.net/)

Protein	pI	Charge at pH 7.5	Amino acid sequence
NPM1^{WT}	4.78	-24.1	GSMEDSMDMDMSPLRPQNYLFGCELKADKDYHFKVDNDENEHQLSLRTVSLGAGAKD<br>ELHIVEAEAMNYEGSPIKVTLATLKMSVQPTVSLGGFEITPPVVLRLKCGSGPVHISGQHLV<br>AVEEDAESEDEEEEDVKLLSISGKRSAPGGGSKVPQKKVKLAADEDDDDDDEEDDDEDD<br>DDDDFDDEEAEEKAPVKKSIRDTPAKNAQKSNQNGKDSKPSSTPRSKGQESFKKQEKTPK<br>TPKGPSSVEDIKAKMQASIEKGGSLPKVEAKFINYVKNCFRMTDQEAIQDLWQWRKSL
NPM1^{N240}	4.64	-26.0	GPLGSMEDSMDMDMSPLRPQNYLFGCELKADKDYHFKVDNDENEHQLSLRTVSLGAG<br>AKDELHIVEAEAMNYEGSPIKVTLATLKMSVQPTVSLGGFEITPPVVLRLKCGSGPVHISG<br>QHLVAVEEDAESEDEEEEDVKLLSISGKRSAPGGGSKVPQKKVKLAADEDDDDDDEEDD<br>DEDDDDDDFDDEEAEEKAPVKKSIRDTPAKNAQKSNQNGKDSKPSSTPRSKGQESFKKQ<br>EKTPKTPKG
NPM1^{N188}	4.24	-37.0	GSMEDSMDMDMSPLRPQNYLFGCELKADKDYHFKVDNDENEHQLSLRTVSLGAGAKD<br>ELHIVEAEAMNYEGSPIKVTLATLKMSVQPTVSLGGFEITPPVVLRLKCGSGPVHISGQHLV<br>AVEEDAESEDEEEEDVKLLSISGKRSAPGGGSKVPQKKVKLAADEDDDDDDEEDDDEDD<br>DDDDFDDEEAEE
NPM1^{mutA3}	8.17	+1.9	GSHMEDSMDMDMSPLRPQNYLFGCELKADKDYHFKVDNDENEHQLSLRTVSLGAGAK<br>DELHIVEAEAMNYEGSPIKVTLATLKMSVQPTVSLGGFEITPPVVLRLKCGSGPVHISGQHL<br>VAVEEDAESEDEEEEDVKLLSISGKRSAPGGGSKVPQKKVKLAAGGSGGSGGSGGSGGSG<br>GSGGSGGSGGSGKAPVKKSIRDTPAKNAQKSNQNGKDSKPSSTPRSKGQESFKKQEKTP<br>KTPKGPSSVEDIKAKMQASIEKGGSLPKVEAKFINYVKNCFRMTDQEAIQDLWQWRKSL
NPM1^{mutB1}	4.6	-30.0	GSHMEDSMDMDMSPLRPQNYLFGCELKADKDYHFKVDNDEEHQLSLRTVSLGAGAKD<br>ELHIVEAEAMNYEGSPIKVTLATLKMSVQPTVSLGGFEITPPVVLRLKCGSGPVHISGQHLV<br>AVEEDAESEDEEEEDVALLSISGARSAPGGGSAVPQAAVALAADEDDDDDDEEDDDEDD<br>DDDDFDDEEAEEKAPVKKSIRDTPAKNAQKSNQNGKDSKPSSTPRSKGQESFKKQEKTPK<br>TPKGPSSVEDIKAKMQASIEKGGSLPKVEAKFINYVKNCFRMTDQEAIQDLWQWRKSL
NPM1^{mutB2}	4.39	-37.0	GSHMEDSMDMDMSPLRPQNYLFGCELKADKDYHFKVDNDENEHQLSLRTVSLGAGAK<br>DELHIVEAEAMNYEGSPIKVTLATLKMSVQPTVSLGGFEITPPVVLRLKCGSGPVHISGQHL<br>VAVEEDAESEDEEEEDVKLLSISGKRSAPGGGSKVPQKKVKLAADEDDDDDDEEDDDED<br>DDDDDFDDEEAEEAAPVAASIRDTPAANAQASNQNGADSAPSSTPRSAGQESFAAQEA<br>TPATPAGPSSVEDIKAKMQASIEKGGSLPKVEAKFINYVKNCFRMTDQEAIQDLWQWRK<br>SL
NPM1^{OD}	5.24	-7.9	GSHMEDSMDMDMSPLRPQNYLFGCELKADKDYHFKVDNDENEHQLSLRTVSLGAGAK<br>DELHIVEAEAMNYEGSPIKVTLATLKMSVQPTVSLGGFEITPPVVLRLKCGSGPVHISGQHL<br>VAVE
NPM1^{CTD}	8.78	+1.7	GSHPSSVEDIKAKMQASIEKGGSLPKVEAKFINYVKNCFRMTDQEAIQDLWQWRKSL
NPM1^{IDR}	4.51	-19.2	GSHMWEEDAESEDEEEEDVKLLSISGKRSAPGGGSKVPQKKVKLAADEDDDDDDEEDD<br>DEDDDDDDFDDEEAEEKAPVKKSIRDTPAKNAQKSNQNGKDSKPSSTPRSKGQESFKKQ<br>EKTPKTPKG
IDR^{188}	3.78	-30.2	EEDAESEDEEEEDVKLLSISGKRSAPGGGSKVPQKKVKLAADEDDDDDDEEDDDEDDDD<br>DDFDDEEAEE
SURF6-N	10.26	+21.0	GSHMASLLAKDAYLQSLAKKICSHSAPEQQARTRAGKTQGSETAGPPKKKRKKTQKKFRK<br>REEKAAEHKAKSLGEKSPAASGARRPEAAKEEAAWASSSAGNPADGLATEPESVFALDVL<br>RQRLHEKIQEARGQGSAKELSPAALEKRRRRKQERDRKKRKRKELRAKEKARKAEEATEA<br>QEVVE

Supplementary Table 2 Best-fit values and estimates of the two-dimensional size-and-shape, *c(s, f/f*$_0$*)*, distribution analyses of NPM1^{WT}, NPM1^{CTD}, NPM1^{N240} and NPM1^{N188} in 10 mM Tris pH 7.5, 150 mM NaCl, 2 mM DTT buffer at 20 °C, Rayleigh interference optical data.

**Sample**	**µM** [a]	s_w **(Svedberg)**[b]	$S_{20,w}$ **(Svedberg)**[c]	**M (kDa)**[d]	**(f/f_{0w})**[e]	**Rs (nm)**[f]
NPM1	47.37	6.74 (79%)	7.00 (79%)	145,112	1.50	4.96
		7.50 (17%)	7.76 (17%)	289,352	2.05	8.88
NPM1^{CTD}	51.25	0.86 (92%)	0.89 (92%)	6,252	1.36	1.66
NPM1^{N240}	34.38	5.68 (92%)	5.87 (92%)	149,784	1.81	6.35
NPM1^{N188}	35.53	4.75 (91%)	4.91 (91%)	118,297	1.87	6.05

[a] Total concentration of sample (µM).
[b] Weight-average sedimentation coefficient s_w calculated from the 2D *c(s, f/f*$_0$*)* model with percentage protein amount of total protein in parenthesis.
[c] Standard sedimentation coefficient ($s_{20,w}$-value) in water at 20 °C.
[d] Estimated molar mass calculated from (s_w, f/f_{0w}) from the 2D *c(s, f/f*$_0$*)* model.
[e] Weight-average frictional ratio (f/f_{0w}) calculated from the 2D *c(s, f/f*$_0$*)* model.
[f] Stokes Radius (nm).

Supplementary Table 3 R_H values of 30µM ^{15}N NPM1^{IDR} in the presence or absence of excess NPM1 domains, as derived from ^{15}N-filtered NMR diffusion experiments.

	R_H (nm)	error
30µM ^{15}N NPM1^{IDR}	2.30	0.16
+ 550µM NPM1^{OD}	2.39	0.18
550µM NPM1^{IDR}	3.02	0.25
+ 550µM NPM1^{CTD}	2.58	0.25

Supplementary Table 4 Primers used in this study.

Mutation	Primer sequence
C21T	Forward 5' CCAGAACTATCTTTTCGGTACTGAACTAAAGGCCGAC 3'<br>Reverse 5' GTCGGCCTTTAGTTCAGTACCGAAAAGATAGTTCTGG 3'
C104T	Forward: 5' GGTCTTAAGGTTGAAGACTGGTTCAGGGCCAGTG 3'<br>Reverse: 5' CACTGGCCCTGAACCAGTCTTCAACCTTAAGACC 3'
S125C	Forward 5'CTTAGTAGCTGTGGAGGAAGATGCAGAGTGCGAAGATGAAGAGGAGGAGGATGTGAAACT 3'<br>Reverse 5'AGTTTCACATCCTCCTCCTCTTCATCTTCGCACTCTGCATCTTCCTCCACAGCTACTAAG 3'
IDR	Forward 5′ AATTAACATATGTGGGAAGAAGACGCTGAATCTGAAGACGAA 3′<br>Reverse 5′ TTAATTGTCGACTTAACCTTTCGGGGTTTTCGGGGTTTTTT 3′
N188	Forward 5' GAAGCTGAAGAATAAGCGCCAGTGAAGAAATCTATACGA 3'<br>Reverse 5' TCGTATAGATTTCTTCACTGGCGCTTATTCTTCAGCTTC 3'
OD	Forward 5′ CCTACCCATATGGAAGACTCGATG 3′<br>Reverse 5′ GCTAGTTATTGCTCAGCGG 3′
CTD	Forward 5′ AATTAACATATGCCGTCTTCTGTTGAAGACATCAAAGC 3′<br>Reverse 5′ AATTAAGTCGACTTACAGAGATTTACGCCACTGC 3′
SURF6-N	Forward 5′CATATGGCCTCTCTACTCGCCAAGGACGCCTAC 3′<br>Reverse 5′GTCGACTTACTCCACCACCTCCTGGGCCTCCGTGG 3′

Supplementary Note 1

The trend in the FRET efficiency (E_{FRET}) histograms of dual-labeled NPM1$^{125-275}$ indicates that there is an expansion in the protein as a function of increasing salt concentration, as revealed in a shift in overall population distribution to lower E_{FRET} values. Our previous work has shown that under the present buffer condition, the specific amount of changes in the [NaCl] has no significant effect on the Alexa488/Alexa594 dye photo-physical properties[1], therefore we conclude that the observed changes are due to alterations in the polypeptide chain.

A detailed analysis of the smFRET histograms clearly reveals broad peaks beyond shot-noise statistics (Fig. 2e). While there could be multiple factors contributing towards this broadening, such as background noise, dye photo-physical processes including bleaching and blinking, non-ideality in observation volume overlap, and others[2, 3], conformational heterogeneity may be a major source. A significant source for the peak broadening could be that multiple structures are being populated by the protein under these conditions. Moreover, it should be noted that if the structures interconvert on a timescale much faster than the 500 µs experimental integration time, the smFRET data would be time-averaged over the different structures, and thus result in a narrower peak with a weighted-average peak position[2, 4]. Therefore, the data indicate that these structures must be interconverting on a timescale slower or similar to that of the experimental data collection. To test for a change in peak width, we ran experiments at an integration time as low as 200 µs, with no significant difference in peak width. We also tested for a peak threshold difference, with no change in peak width over increasing threshold, suggesting a non-significant contribution by background noise.

To further analyze this aspect, we tried arbitrarily fitting NPM1 histograms with two overlapping peaks, one at high E_{FRET} (0.87) and the other at intermediate E_{FRET} (0.55) using Gaussian model. This is shown is SI Fig-3a. With increasing salt concentration, the population density of the species with lower E_{FRET} increased from ~ 55% at 50 mM NaCl to 80% 300 mM NaCl. Therefore, both one-peak and tow-peak fits of our smFRET data support the expansion of the protein at higher salt, entirely consistent with the SAXS data.

Supplementary References

1. Banerjee, P.R., Mitrea, D.M., Kriwacki, R.W. & Deniz, A.A. Asymmetric Modulation of Protein Order-Disorder Transitions by Phosphorylation and Partner Binding. *Angewandte Chemie* **55**, 1675-1679 (2016).
2. Mukhopadhyay, S., Krishnan, R., Lemke, E.A., Lindquist, S. & Deniz, A.A. A natively unfolded yeast prion monomer adopts an ensemble of collapsed and rapidly fluctuating structures. *Proc Natl Acad Sci U S A* **104**, 2649-2654 (2007).
3. Ferreon, A.C., Gambin, Y., Lemke, E.A. & Deniz, A.A. Interplay of alpha-synuclein binding and conformational switching probed by single-molecule fluorescence. *Proceedings of the National Academy of Sciences of the United States of America* **106**, 5645-5650 (2009).
4. Gopich, I. & Szabo, A. Theory of photon statistics in single-molecule Förster resonance energy transfer. *The Journal of chemical physics* **122**, 014707 (2005).
