# Supplementary material for: Self-interaction of NPM1 modulates multiple mechanisms of liquid–liquid phase separation
Source: Nat Commun. 2018 Feb 26;9:842. doi: 10.1038/s41467-018-03255-3 (PMC5827731; doi:10.1038/s41467-018-03255-3)
Supplement: Supplementary file 3 — Description of Additional Supplementary Files [file 41467_2018_3255_MOESM3_ESM.pdf]

Description of Supplementary Files

File Name: Supplementary Movie 1
Description: **NPM1^{WT}:SURF6-N LLPS** Time lapse of 20 µM NPM1^{WT} and 20 µM SURF6-N droplets in 10 mM Tris, 150 mM NaCl, 2 mM DTT pH 7.5 buffer “rain” from the bulk solution down onto the coverslip, coalesce, fuse and relax. Droplets are visualized by fluorescence confocal microscopy, following fluorescence of SURF6-N labeled with AlexaFluor647.

File Name: Supplementary Movie 2
Description: **NPM1^{N240}:SURF6-N LLPS** Time lapse of 20 µM NPM1^{N240} and 20 µM SURF6-N droplets in 10 mM Tris, 150 mM NaCl, 2 mM DTT pH 7.5 buffer “rain” from the bulk solution down onto the coverslip, coalesce, fuse and relax. Droplets are visualized by fluorescence confocal microscopy, following fluorescence of SURF6-N labeled with AlexaFluor647.

File Name: Supplementary Movie 3
Description: **NPM1^{N188}:SURF6-N LLPS** Time lapse of 20 µM NPM1^{N188} and 20 µM SURF6-N droplets in 10 mM Tris, 150 mM NaCl, 2 mM DTT pH 7.5 buffer “rain” from the bulk solution down onto the coverslip, coalesce, fuse and relax. Droplets are visualized by fluorescence confocal microscopy, following fluorescence of SURF6-N labeled with AlexaFluor647.
